# Analysis and validation of diagnostic biomarkers and immune cell infiltration characteristics in pediatric sepsis by integrating bioinformatics and machine learning

**DOI:** 10.1007/s12519-023-00717-7

**Published:** 2023-04-28

**Authors:** Wen-Yuan Zhang, Zhong-Hua Chen, Xiao-Xia An, Hui Li, Hua-Lin Zhang, Shui-Jing Wu, Yu-Qian Guo, Kai Zhang, Cong-Li Zeng, Xiang-Ming Fang

**Affiliations:** 1 Department of Anesthesiology and Intensive Care, School of Medicine, The First Affiliated Hospital, Zhejiang University, QingChun Road 79, Hangzhou, 310003 China; 2 Department of Anesthesiology, Shaoxing People’s Hospital (Shaoxing Hospital, Zhejiang University School of Medicine), Shaoxing, 312000 China; 3 Department of Anesthesiology, Vagelos College of Physicians and Surgeons, Columbia University, New York, NY USA

**Keywords:** Paediatric sepsis, Bioinformatics, Machine-Learning, Biomarkers, Immune cell infiltration

## Abstract

**Background:**

Pediatric sepsis is a complicated condition characterized by life-threatening organ failure resulting from a dysregulated host response to infection in children. It is associated with high rates of morbidity and mortality, and rapid detection and administration of antimicrobials have been emphasized. The objective of this study was to evaluate the diagnostic biomarkers of pediatric sepsis and the function of immune cell infiltration in the development of this illness.

**Methods:**

Three gene expression datasets were available from the Gene Expression Omnibus collection. First, the differentially expressed genes (DEGs) were found with the use of the *R* program, and then gene set enrichment analysis was carried out. Subsequently, the DEGs were combined with the major module genes chosen using the weighted gene co-expression network. The hub genes were identified by the use of three machine-learning algorithms: random forest, support vector machine-recursive feature elimination, and least absolute shrinkage and selection operator. The receiver operating characteristic curve and nomogram model were used to verify the discrimination and efficacy of the hub genes. In addition, the inflammatory and immune status of pediatric sepsis was assessed using cell-type identification by estimating relative subsets of RNA transcripts (CIBERSORT). The relationship between the diagnostic markers and infiltrating immune cells was further studied.

**Results:**

Overall, after overlapping key module genes and DEGs, we detected 402 overlapping genes. As pediatric sepsis diagnostic indicators, CYSTM1 (AUC = 0.988), MMP8 (AUC = 0.973), and CD177 (AUC = 0.986) were investigated and demonstrated statistically significant differences (*P* < 0.05) and diagnostic efficacy in the validation set. As indicated by the immune cell infiltration analysis, multiple immune cells may be involved in the development of pediatric sepsis. Additionally, all diagnostic characteristics may correlate with immune cells to varying degrees.

**Conclusions:**

The candidate hub genes (CD177, CYSTM1, and MMP8) were identified, and the nomogram was constructed for pediatric sepsis diagnosis. Our study could provide potential peripheral blood diagnostic candidate genes for pediatric sepsis patients.

**Supplementary Information:**

The online version contains supplementary material available at 10.1007/s12519-023-00717-7.

## Introduction

Sepsis, a potentially fatal illness, is a significant public health concern because of its association with an aberrant immune response to infections and severe organ failure [1]. There are an estimated 22 instances of pediatric sepsis per 100,000 person-years and 2202 cases of neonatal sepsis per 100,000 live births worldwide, totaling 1.2 million cases of pediatric sepsis annually [2]. Additionally, the case-fatality rate for pediatric sepsis following diagnosis is expected to be 25% [3]. Most children who die from sepsis have refractory shock and/or multiple organ dysfunction syndrome, and many die during the first 48–72 h of treatment [4, 5]. To maximize outcomes for children with sepsis, early detection, adequate resuscitation, and care are crucial.

There are accumulating evidences that the systemic immune response plays a crucial role in the etiology and progression of sepsis [6–8]. Early in sepsis, the immune response is primarily proinflammatory and helpful in eliminating pathogens [9]. Suppression of the immune response, as seen by a decrease in the function and number of immune cells, is a primary hallmark of progressive sepsis [10]. A weakened immune system in the host may also be intimately associated with the poor prognosis of sepsis [11, 12]. In addition, an increasing number of studies have revealed that novel immunological biomarkers not only have the potential to serve as possible predictors of the prognosis of sepsis but also have the ability to act as prospective targets for immunotherapy of sepsis [13, 14].

High-throughput sequencing is a useful method for investigating changes in illness gene expression and identifying possible disease-related genes to discover new diagnostic and therapeutic approaches [15]. The level of gene expression can indicate the condition of a variety of disorders, including pediatric sepsis, and is a vital indicator for basic diagnosis [16–18]. Using either supervised or unsupervised techniques, machine-learning algorithms have shown great promise in analyzing the underlying relationship of high-dimensional data [19, 20]. Furthermore, machine learning is useful for assessing high-dimensional transcriptome data and locating genes that are of biological significance [21, 22]. Recently, early diagnosis and clinical treatment of pediatric sepsis are more likely to be successful now that common disease-associated variations in the population have been identified by large-scale genome-wide association studies (GWAS) and meta-analyses [23, 24].

Compared to previous studies [25], we not only integrated multiple high-throughput sequencing data of pediatric sepsis for analysis but also, more importantly, used machine learning to screen characteristic genes for the first time. Immune cell infiltration refers to the migration of immune cells to diseased tissues. Furthermore, the relationships between diagnostic markers and invading immune cells were investigated to acquire a deeper understanding of the molecular immunological mechanisms involved in the development of pediatric sepsis. This was done to acquire a deeper understanding of the molecular immunological mechanisms involved in the development of pediatric sepsis.

## Methods

### Gene expression profiles

Accessing the Gene Expression Omnibus (GEO) database (https://www.ncbi.nlm.nih.gov/geo/), which is a public collection of high-throughput gene expression data, chips, and microarrays, was how the information was collected [26]. We searched the GEO database with the keywords “Pediatric sepsis” [MeSH Terms] AND “Homo sapiens” [porgn: txid9606] AND “Expression profiling by array” [All Fields]. The following were included among the criteria used for screening: the microarray datasets referred to profiles of genome-wide gene expression in blood. The microarray datasets contained samples from pediatric sepsis and samples from a healthy state. None of the included samples were associated with any other diseases. The sample size of both the pediatric sepsis group and the normal group was greater than 10. In the end, three different gene expression datasets were analyzed to complete the in-depth investigation. Supplementary Table 1 contains information regarding these different gene expression datasets. GSE26378 [27] and GSE26440 [28] were used as the analysis set, and GSE13904 [29] was used as the validation set.

### Identification of the differentially expressed genes

Gene expression profile files were generated by normalizing the data and correcting the expression value in batches using the "sva" package [30]. This was done after GSE26378 and GSE26440 were combined. The LIMMA package [31] was utilized to identify DEGs between the pediatric sepsis group and the control group, and a volcano plot was produced to highlight the differential expression of DEGs. Adjusted *P* values were looked at in GEO to account for the possibility of false-positive outcomes. An adjusted *P* value < 0.05 and |log2FC|> 0.5 were considered to be the cutoffs for DEGs. Using R software's pheatmap package, a heatmap was generated based on the DEGs that had been screened.

### Gene set enrichment analysis (GSEA)

To provide a more intuitively obvious picture of the gene expression level of highly enriched functional pathways, GSEA [32] was conducted using the R programming language. There was statistical significance when the adjusted *P* value was less than 0.05.

### Enhancement of functionality

Functional enrichment was applied to the data to verify the likely functions of potential targets. Gene ontology, often known as GO, is a well-known method for ascribing functions to genes, in particular molecular functions (MF), biological pathways (BP), and cellular components (CC) [33]. KEGG enrichment analysis can be used to investigate not only the activities of genes but also the high-level genomic information connected to those functions [34]. To further understand the carcinogenic significance of target genes, we used R's "GOplot" package [35] and "cluster profiler" to analyze the GO function of candidate mRNAs and to enhance KEGG pathways.

### Protein‒protein interaction (PPI) network construction

In addition to being aware of the fundamental roles that proteins play in cellular processes, it is essential to be familiar with the associated activities that proteins play. Functional connections between differentially expressed genes (DEGs) and other genes were annotated with the help of the Search Tool for the Retrieval of Interacting Genes (STRING) online database (http://string-db.org) [36]. The PPI network was constructed using only those interactions that had been empirically validated and had a total score that was higher than 0.4.

### Construction of coexpressed gene modules

The weighted gene co-expression network (WGCNA) approach is helpful in conducting research on the expression of gene sets. The WGCNA R package was utilized throughout the following primary phases for the construction and modularization of distinct gene networks at various stages. The samples were organized into clusters to identify any potentially significant outliers that may have been present. Then, automated network systems were utilized to establish co-expression networks. Hierarchical clustering and dynamic tree cutting function detection were both utilized by the modules. To establish a connection between modules and clinical features, estimates of module membership (MM) and gene significance (GS) were made. The modules that had the highest Pearson module membership correlation (MM) and a *P* absolute value of 0.05 were chosen to be the hub modules. The values of MM > 0.8 and GS > 0.2 were indicative of a highly connected module and clinical relevance, respectively. In preparation for more research, the gene information for the associated module was given [37].

### Screening and validation of diagnostic markers

New and important biomarkers for pediatric sepsis were screened using three machine-learning algorithms: random forests (RF) [38–40], least absolute shrinkage and selection operator (LASSO) logistic regression [41, 42], and support vector machine-recursive feature elimination (SVM-RFE) [43]. The "randomForest" *R* package in *R* was used to implement the random forest technique in this study. This study carried out LASSO logistic regression investigation with the *R* package “glmnet” [44], and minimal lambda was considered optimal. In our study, the selection of optimization parameters was cross-verified by a factor of 10, and the partial likelihood deviation met the minimum criteria. The genes that have traits in common with more than one of the three classification models discussed before were then selected for additional study. The validation set for the complete analysis of the usefulness of significant biomarkers will be the dataset from GSE13904. It was evaluated based on the study of receiver operating characteristic (ROC) curves, and the area under the curve (AUC) was calculated to measure the predictive capability of the algorithms. Statistical significance was determined by a two-tailed test with a *P* value of less than 0.05.

### Evaluation and correlation analysis of infiltration-related immune cells

Utilizing the website CIBERSORT, 22 types of immune cell matrices were filtered. *P* < 0.05 demonstrated that the immune cell infiltration matrix was acquired [45]. The Spearman association between unique diagnostic markers and immune invading cells was analyzed using the "ggstatsplot" and "ggplot2" packages to illustrate the results.

## Results

### Screening of DEGs in pediatric sepsis

**Fig. 1:**
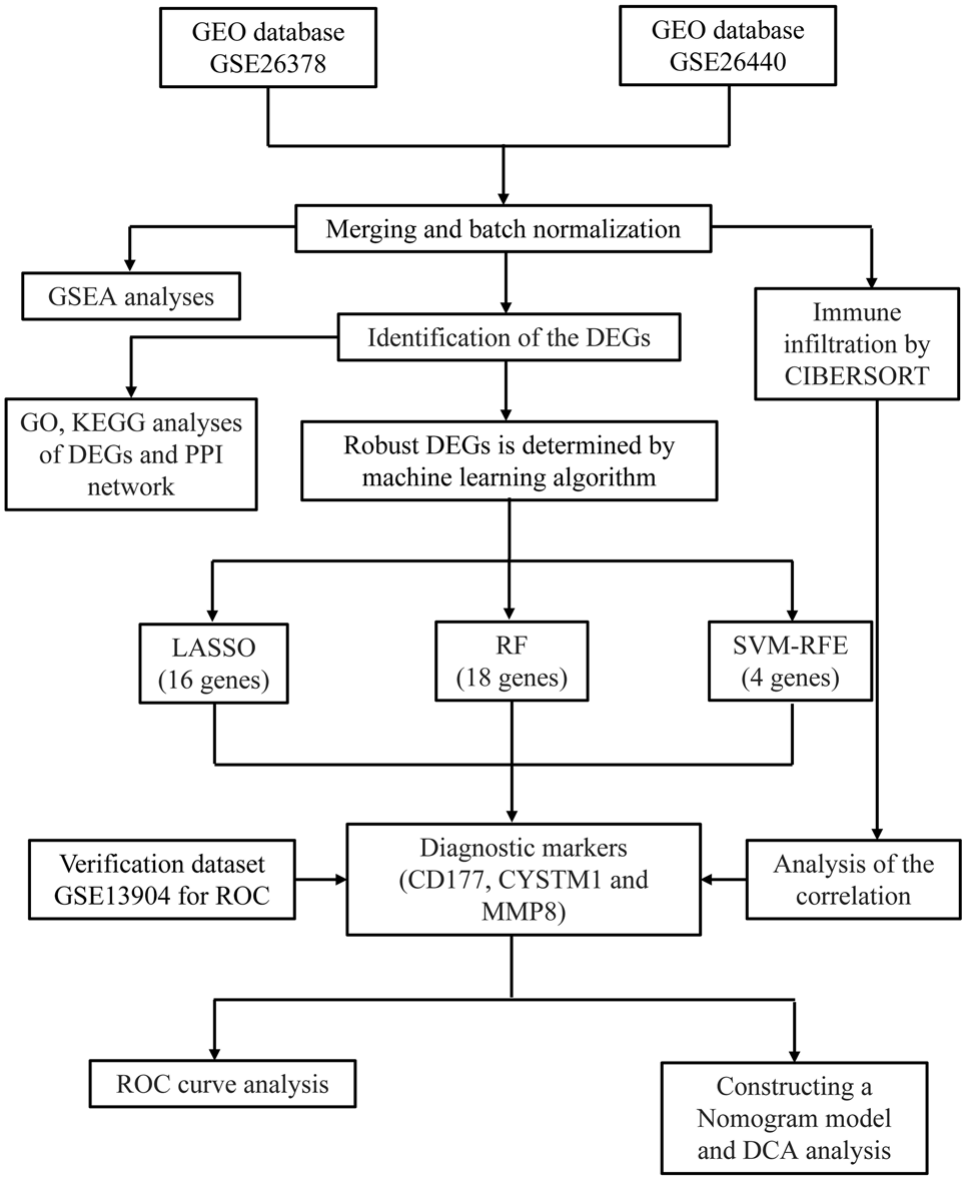
The flowchart depicting the investigation procedure. *GEO* gene expression omnibus, *GSEA* gene set enrichment analysis, *CIBERSORT* cell-type identification by estimating relative subsets of RNA transcripts, *DEGs* differentially expressed genes, *GO* gene ontology, *KEGG* Kyoto Encyclopedia of Genes and Genomes, *PPI* protein‒protein interaction, *LASSO* Least absolute shrinkage and selection operator, *RF* random forest, *SVM-RFE* support vector machine-recursive feature elimination, *ROC* receiver operating characteristic curve, *DCA* decision curve analysis

The clinical characteristics of the two groups of patients are presented in Supplementary Table 2. The process of the research is depicted in Fig. 1. There were 556 DEGs, including 381 upregulated and 175 downregulated genes (Supplementary Fig. 1a, b).

### GSEA

GSEA was carried out on both pediatric patients with sepsis and healthy control subjects to investigate the biological signaling pathway. The top five terms identified by HALLMARK analysis are displayed (Supplementary Fig. 2a). Coagulation, complement, IL6–JAK–STAT3 signaling, inflammatory response and TNFα signaling via NFκB were considerably enriched in the patients with pediatric sepsis (*P* adjusted < 0.05).

### Functional enrichment analysis of DEGs

We conducted functional analysis to gain a deeper understanding of the biological functions of the DEGs. The results of DO analysis revealed that these DEGs were linked to lung disease, arteriosclerosis, hepatitis, atherosclerosis, arteriosclerotic cardiovascular disease, bacterial infectious disease, primary bacterial infectious disease, obstructive lung disease, tuberculosis and bronchial disease (Supplementary Fig. 2b). GO enrichment analysis showed that DEGs have immune response-regulating signaling pathways, activation of the immune response, positive regulation of cytokine production, leukocyte-mediated immunity, T-cell activation and myeloid leukocyte activation (Supplementary Table 3 and Supplementary Fig. 2c). KEGG analysis was associated with hematopoietic cell lineage, *Staphylococcus aureus* infection, Th1 and Th2 cell differentiation and Th17 cell differentiation (Supplementary Fig. 2d). Supplementary Fig. 2e illustrates the PPI network.

### Identification of co-expression gene modules in pediatrics sepsis

In pediatric sepsis datasets, we used WGCNA to locate gene modules that were coexpressed by many genes. First, samples from both datasets were grouped into two groups, pediatric sepsis group and normal group, with no outliers observed (Supplementary Fig. 3a). Then, based on scale independence of > 0.8, 13 was selected as the soft thresholding power β to ensure biologically significant scale-free network (Supplementary Fig. 3b, c). Through hierarchical clustering analysis and dynamic branch cut methods for gene dendrograms, genes were grouped into 12 modules (Supplementary Fig. 3d, e). The lightcyan, blue and lightgreen module were significantly associated with pediatrics sepsis and selected for further analysis (Supplementary Fig. 3f, *P* < 0.05). The scatter plot is shown in Supplementary Fig. 3g, Supplementary Fig. 3h and Supplementary Fig. 3i. A total of 1582 genes were significantly associated with pediatrics sepsis gene significance and module membership.

### Screening and validation of diagnostic markers

**Fig. 2:**
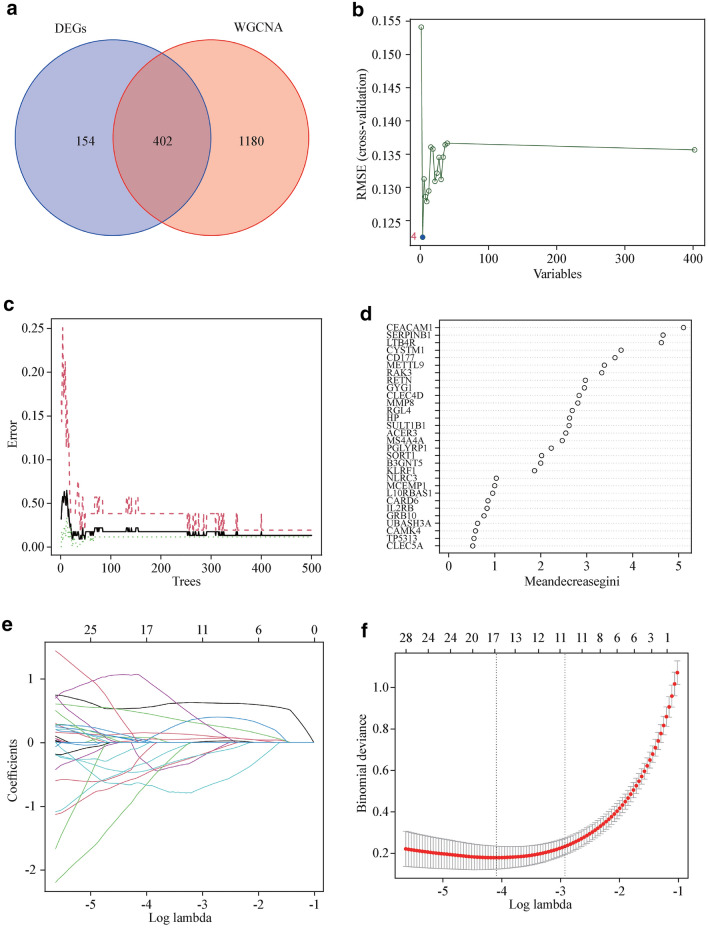
Detection of diagnostic markers using a thorough method. **a** Venn diagram of key module genes versus DEGs; **b** based on SVM-RFE to screen biomarkers; **c**, **d** Based on RF algorithm to screen biomarkers; **e** Different colors represent different genes; **f** LASSO logistic regression algorithm to screen diagnostic markers. *DEGs* differentially expressed genes, *WGCNA* weighted gene co-expression network, *SVM-RFE* support vector machine-recursive feature elimination, *RF* random forest, *LASSO* least absolute shrinkage and selection operator

**Fig. 3:**
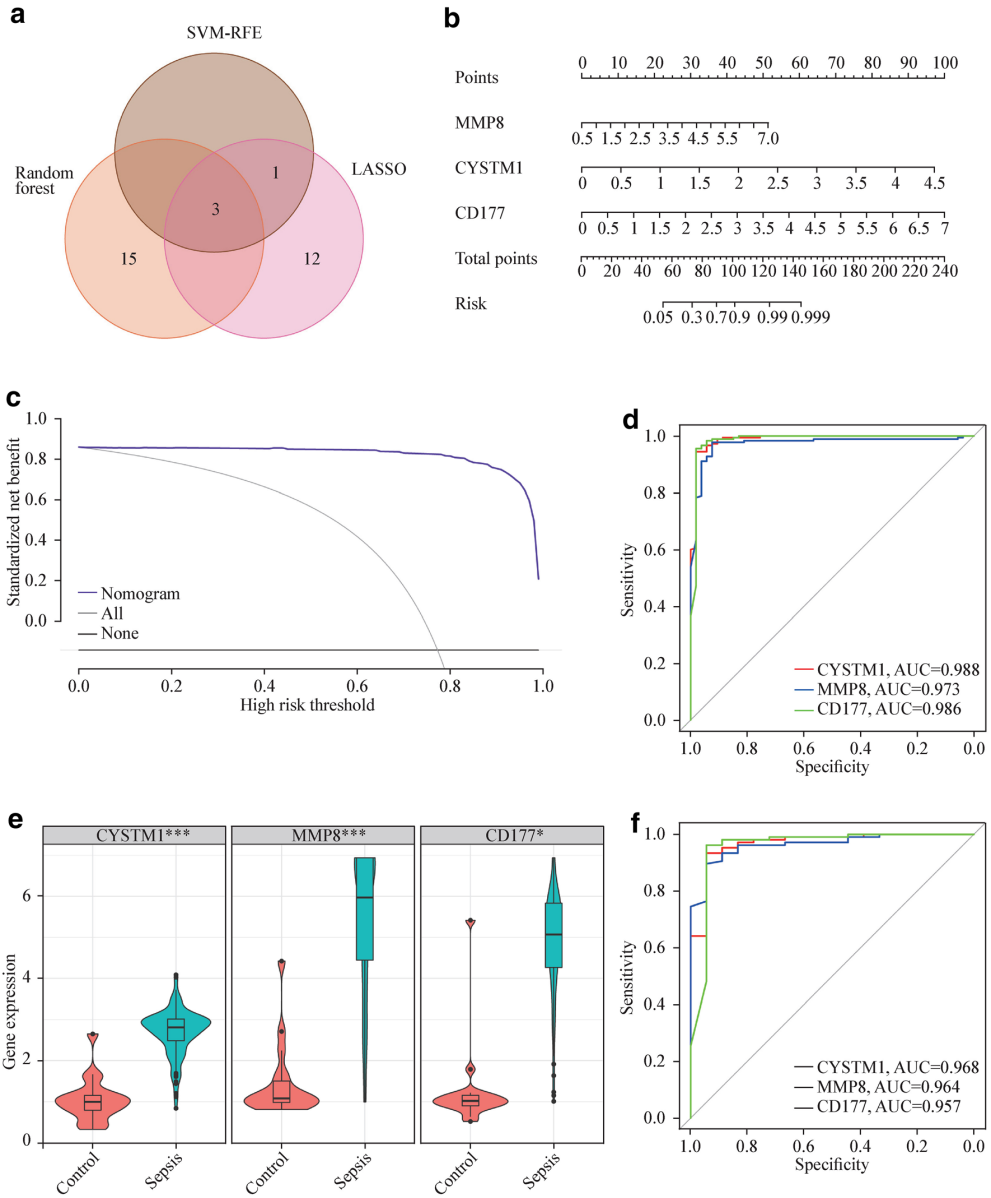
Hub genes for pediatric sepsis diagnosis. **a** Venn diagram showed the intersection of diagnostic markers obtained by the three algorithms; **b** Nomogram is used to predict the occurrence of pediatric sepsis; **c** DCA curves **d** the ROC curve of the diagnostic efficacy verification; **e** Boxplot showed the expression of hub genes between pediatric sepsis group and control group in validation set; **f** The ROC curve of the diagnostic efficacy verification in validation set. *SVM-RFE* support vector machine-recursive feature elimination, *RF* random forest, *LASSO* least absolute shrinkage and selection operator, *AUC* area under curve, *ROC* receiver operating characteristic curve, *DCA* decision curve analysis

By utilizing a Venn diagram to compare the overlapping regions of DEGs and key module genes, we were able to identify 402 overlapping gene regions (Fig. 2a). We used three machine-learning algorithms to identify feature genes: SVM-RFE (Fig. 2b); RF in combination with feature selection was used to determine the connection between the error rate, the number of classification trees, and the 18 genes in descending order of relative relevance (Fig. 2c, d); and LASSO regression analysis was used to select 16 predicted genes from among the statistically significant univariate variables (Figs. 2e, f). The three algorithms identified CYSTM1, MMP8, and CD177 as genes with overlap (Fig. 3a). Using the rms package, we developed nomogram models for diagnosing pediatric sepsis based on the hallmark genes CYSTMI1, MMP8, and CD177 (Fig. 3b). According to the results of the decision curve analysis (DCA), the nomogram model offered a better clinical benefit (Fig. 3c). With AUCs of 0.988, 0.973, and 0.986, respectively, the ROC curves for CYSTM1, MMP8, and CD177 indicated their likelihood as valuable biomarkers (Fig. 3d), indicating that the biological markers had a high predictive value accuracy. In the GSE13904 validation set, the expression of CYSTM1, MMP8, and CD177 was considerably higher in the pediatric sepsis group than in the control group (*P* < 0.01) (Fig. 3e). With AUCs of 0.968, 0.964, and 0.957, respectively, the ROC curves for CYSTM1, MMP8, and CD177 indicated their likelihood as valuable biomarkers in the GSE13904 validation set (Fig. 3f).

### Infiltration of immune cells results

With the CIBERSORT algorithm, in comparison with normal samples, pediatric sepsis samples generally contained a higher proportion of monocytes, M0 macrophages, M1 macrophages, M2 macrophages, resting mast cells, activated mast cells, eosinophils and neutrophils, and naïve B cells, CD8+ T cells, resting CD4+ memory T cells, activated CD4+ memory T cells, Trges, resting NK cells, activated NK cells and resting dendritic cells were relatively lower (*P* < 0.05) (Supplementary Fig. 4a, b). In accordance with the findings of the correlation analysis, CYSTM1, MMP8, and CD177 exhibited a significant degree of connection with a wide variety of immune cells (Supplementary Fig. 5).

## Discussion

In general, sepsis is a dysregulated host response to infectious pathogens and acts as the final common pathway for children who are suffering from a variety of primary and secondary illnesses [46]. Children not only have rapid physiologic development but also have major changes in their immune systems from the time they are born until they enter their teenage years. These changes have an effect on how children react to microorganisms, especially respiratory infections [47]. Current treatment guidelines for sepsis highlight the importance of a strong index of suspicion for early detection of sepsis and prompt administration of antibiotics as critical concepts for improving patient outcomes [48]. Therefore, to improve the prognosis of patients suffering from pediatric sepsis, it is essential to search for a specific diagnostic marker and investigate the patterns of cell infiltration that are associated with pediatric sepsis immune cells. This will allow for a better understanding of how pediatric sepsis affects the immune system. In the current investigation, an effort was made to identify a diagnostic marker that is specific to pediatric sepsis, and an investigation into the effect of the infiltration of immune cells into pediatric sepsis was also carried out.

In this work, attempts were made to identify a diagnostic marker for pediatric sepsis, and the impact of immune cell infiltration on pediatric sepsis was investigated (e.g., coagulation, complement, IL6-JAK-STAT3 signaling, inflammatory response and TNFα signaling via NFκB). Numerous investigations have demonstrated that a substantial proportion of sepsis-related deaths are due to unresolved opportunistic infections and immunosuppressive characteristics [6–8]. Sepsis can be viewed as a race to the grave between infections and the immune response of the host, with pathogens attempting to gain an advantage by impeding various aspects of host immunity [9]. In addition, sepsis has a direct influence on the immune system as a result of the negative changes it causes in the formation, maturation, function, and death of immune cells [49].

Between the pediatric sepsis and the control groups, our research revealed a total of 556 DEGs, with 381 genes being upregulated and 175 genes being downregulated. Subsequent GO enrichment analysis showed that all DEGs were mainly associated with the immune response-regulating signaling pathway, activation of the immune response, positive regulation of cytokine production, leukocyte-mediated immunity, T-cell activation and myeloid leukocyte activation, while KEGG enrichment analysis showed some correlation with hematopoietic cell lineage, *S. aureus* infection, Th1 and Th2 cell differentiation and Th17 cell differentiation. On the basis of these DEGs, we incorporated WGCNA and three machine-learning algorithms to screen and identify pediatric sepsis diagnostic markers. The RF model is an example of a non-parametric technique that can be used to achieve classification while being supervised [38, 39]. RF is inclusive of the decision tree that has been constructed based on the datasets that have been segmented. Within the scope of this study, the training and analysis for one RF classifying model were carried out to locate descriptors that may separate RA from the general sample. LASSO logistic regression is an example of a machine-learning technique; it defines variables by searching for the value that corresponds to the lowest possible likelihood of classification error [41, 42]. SVM recursive feature elimination, also known as SVM-RFE, is a method of machine learning that has numerous applications, including the ranking of features and the selection of the most significant ones for classification [43]. In the end, CYSTM1, MMP8 and CD177 were chosen, and the results of the in-depth verifications showed that they were accurate. Therefore, our prediction accurately represented the practicability displayed by the integration approach. Numerous reports have indicated a definite link between the two hub genes, MMP8 and CD177, and the process of sepsis to some extent. This association was shown to be significant in some cases. Serum MMP8 levels have been shown to be much greater in patients with severe sepsis than in healthy controls, according to research conducted by Wong et al., who found that MMP8 gene expression is overexpressed in children who are experiencing septic shock [29]. It is evident that MMP8 functions as a new regulator of inflammation in sepsis. CD177 is expressed primarily by neutrophils in the acute neutrophil response to an infection via neutrophil adhesion and transendothelial migration, and neutrophil CD177 mRNA levels are enhanced in numerous diseases associated with elevated neutrophil numbers, such as severe sepsis. Although CYSTM1 has not been subjected to considerable research in sepsis until now, it possesses the potential to be an effective novel therapeutic target once further validation has been carried out.

The CIBERSORT program was utilized in the current investigation to analyze the immune infiltration process that occurs during pediatric sepsis. This was done to get a more in-depth understanding of the effects that are brought about by the infiltration of immune cells in pediatric sepsis, which was the motivation behind why this was done. Changes in the infiltration of various immune cells may be associated with the incidence and progression of sepsis in children. NK cells constitute the body's first line of defense against viral infections and the formation of tumors and are also necessary for maintaining the homeostasis of healthy tissues [50]. Boosting the activity of NK cells may enhance the alleviation of viral infections and even severe infection-induced sepsis. Classically activated macrophages (M1) undergo re-programming to become an alternative phenotype called M2 after an initial phase of inflammation. This second phase adds to secondary immune suppression, also known as immunoparalysis, which occurs during sepsis and works against the process of removing infectious organisms to ultimately worsen pathological conditions [51]. Others in the scientific community have observed that neutrophils are necessary for the control of pathogens in the early period of sepsis. It is interesting to note that patients with sepsis have a significant number of circulating neutrophils of varying degrees of maturation. This is because the increased release of immature neutrophils and the delayed death of circulating neutrophils both contribute to this phenomenon [52].

To uncover diagnostic indications for RA, new scientific methodologies such as SVM-RFE, LASSO logistic regression, and the RF algorithm were applied. In addition, CIBERSORT was utilized to examine the invasion of immune cells. Nonetheless, this study has several limitations. The CIBERSORT investigation is consistent with the little genetic information available, which may have been caused by disease-induced diseases, cellular heterogeneity interaction processes, or phenotypic plastic properties. Furthermore, the findings of this research indicate the need for further mining and investigation of previously collected datasets. Although the findings of some earlier studies do not agree with the findings of this analysis, it is imperative that large-scale experiments be carried out to determine whether the findings of this study are reliable.

This study concluded that CYSTM1, MMP8 and CD177 are pediatric sepsis diagnostic indicators. Additionally, the results of this study demonstrated that immune cells may have a role in the onset and progression of pediatric sepsis. CYSTM1, MMP8, and CD177 were also found to have significant connections with a wide variety of immune cell types. It is anticipated that the immune cells mentioned above will have a significant impact on the development of pediatric sepsis. It is also likely that a comprehensive examination of these immune cells will assist in the identification of immunotherapy targets and the optimization of immunomodulatory therapy for patients suffering from pediatric sepsis.

### Supplementary Information

Below is the link to the electronic supplementary material.

Supplementary file1 (PDF 1366 KB)

## Data Availability

All data are available and the correspondent can be contacted if requested.
