# Supplementary material for: Analysis and validation of diagnostic biomarkers and immune cell infiltration characteristics in pediatric sepsis by integrating bioinformatics and machine learning
Source: World J Pediatr. 2023 Apr 28;19(11):1094–103. doi: 10.1007/s12519-023-00717-7 (PMC10533616; doi:10.1007/s12519-023-00717-7)
Supplement: Supplementary file 1 — Supplementary file1 (PDF 1366 KB) [file 12519_2023_717_MOESM1_ESM.pdf]

Supplementary Table 1. Information on microarray datasets obtained from GEO

GEO dataset	Platform	pediatric sepsis	Normal
GSE26378	GPL570	72	21
GSE26440	GPL570	98	32
GSE13904	GPL570	106	18

GEO Gene Expression Omnibus

Supplementary Table 2. The clinical characteristics of the two groups of patients

Characteristics	Pediatrics sepsis	Normal
Number	170	53
Age (Years)	2.3 (0.9-6.1)	2.2 (0.7-4.8)
Number of deaths (%)	29 (17.1)	0 (0)

Supplementary Table 3. GO enrichment analysis of DEGs

Ontology	Term	Description
BP	GO:0002764	immune response-regulating signaling pathway
BP	GO:0002253	activation of immune response
BP	GO:0001819	positive regulation of cytokine production
BP	GO:0002443	leukocyte mediated immunity
BP	GO:0042110	T cell activation
BP	GO:0002274	myeloid leukocyte activation
CC	GO:0070820	tertiary granule
CC	GO:0042581	specific granule
CC	GO:0034774	secretory granule lumen
CC	GO:0060205	cytoplasmic vesicle lumen
CC	GO:0031983	vesicle lumen
CC	GO:0035580	specific granule lumen
MF	GO:0140375	immune receptor activity
MF	GO:0023023	MHC protein complex binding
MF	GO:0030246	carbohydrate binding

MF	GO:0004896	cytokine receptor activity
MF	GO:0023026	MHC class II protein complex binding
MF	GO:0003953	NAD+ nucleosidase activity

DEGs differentially expressed genes, *GO* gene ontology, *BP* biological pathways, *CC* cellular components, *MF* molecular functions, *MHC* major histocompatibility complex, *NAD* nicotinamide adenine dinucleotide.

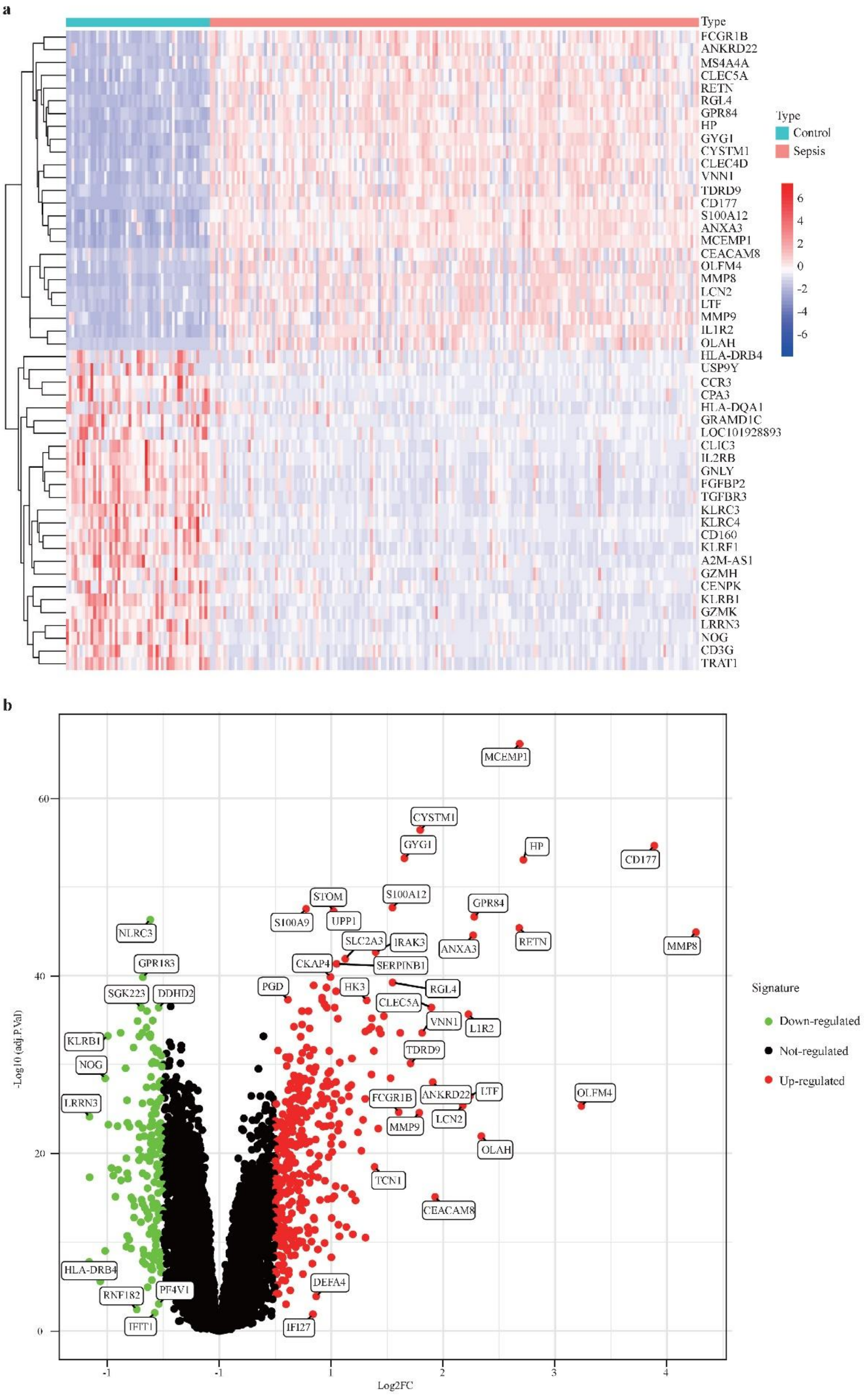

Supplementary Fig. 1. Detection of differentially expressed genes from datasets on pediatric sepsis. (a) A heatmap comparing the genes that are differentially expressed in paediatricpediatric sepsis patients and control patients; (b) Volcano plot of the 556 DEGs. *DEGs* differentially expressed genes, *FC* fold-change.

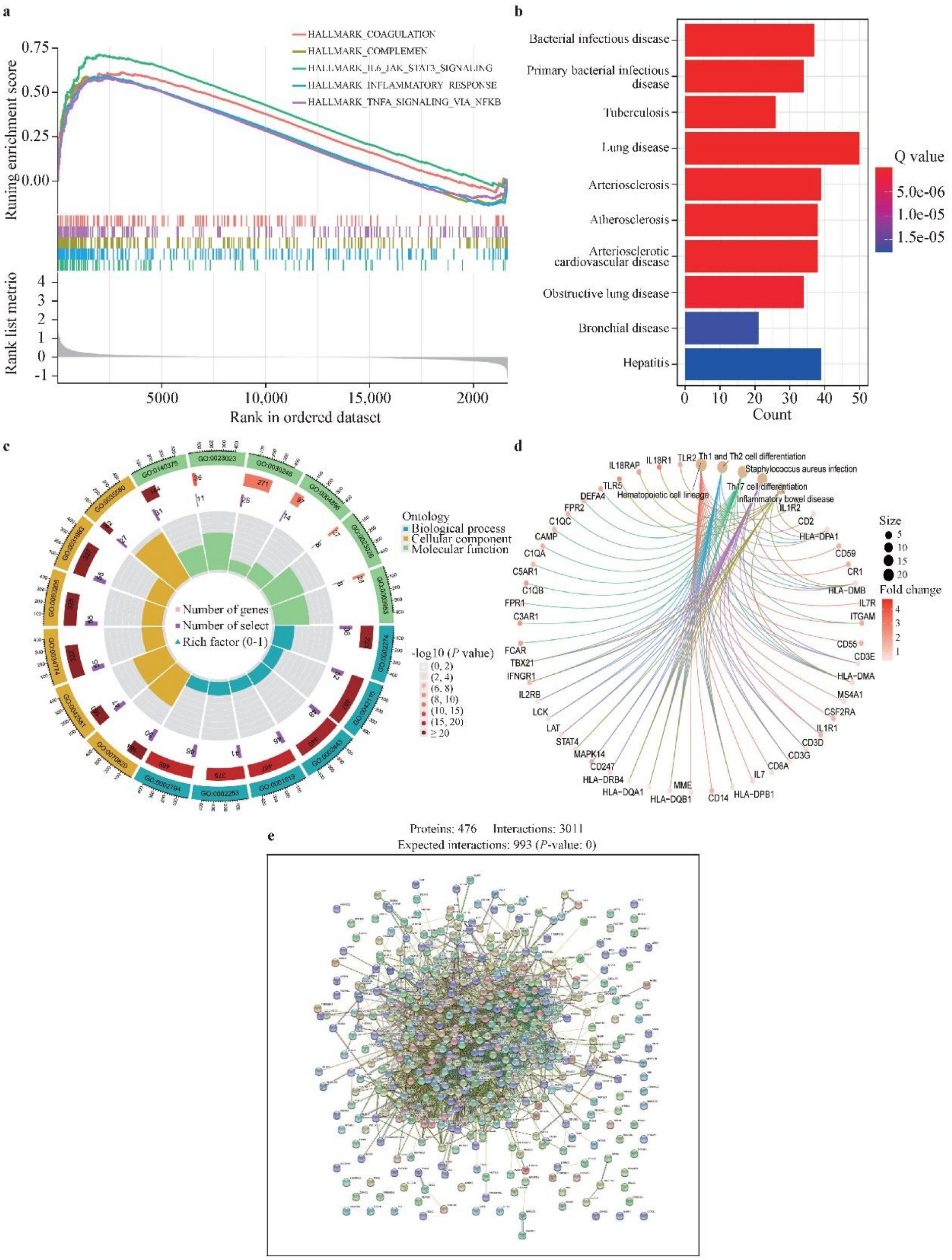

Supplementary Fig. 2. Functional analysis of DEGs. (a) GSEA analysis; (b) DO analysis; (c) GO enrichment analysis; (d) KEGG pathway enrichment analysis; (e) PPI networks. *DEGs* differentially expressed genes, *GSEA* gene set enrichment analysis, *DO* disease ontology, *GO* gene ontology, *KEGG* kyoto encyclopedia of genes and genomes, *PPI* Protein–protein interaction.

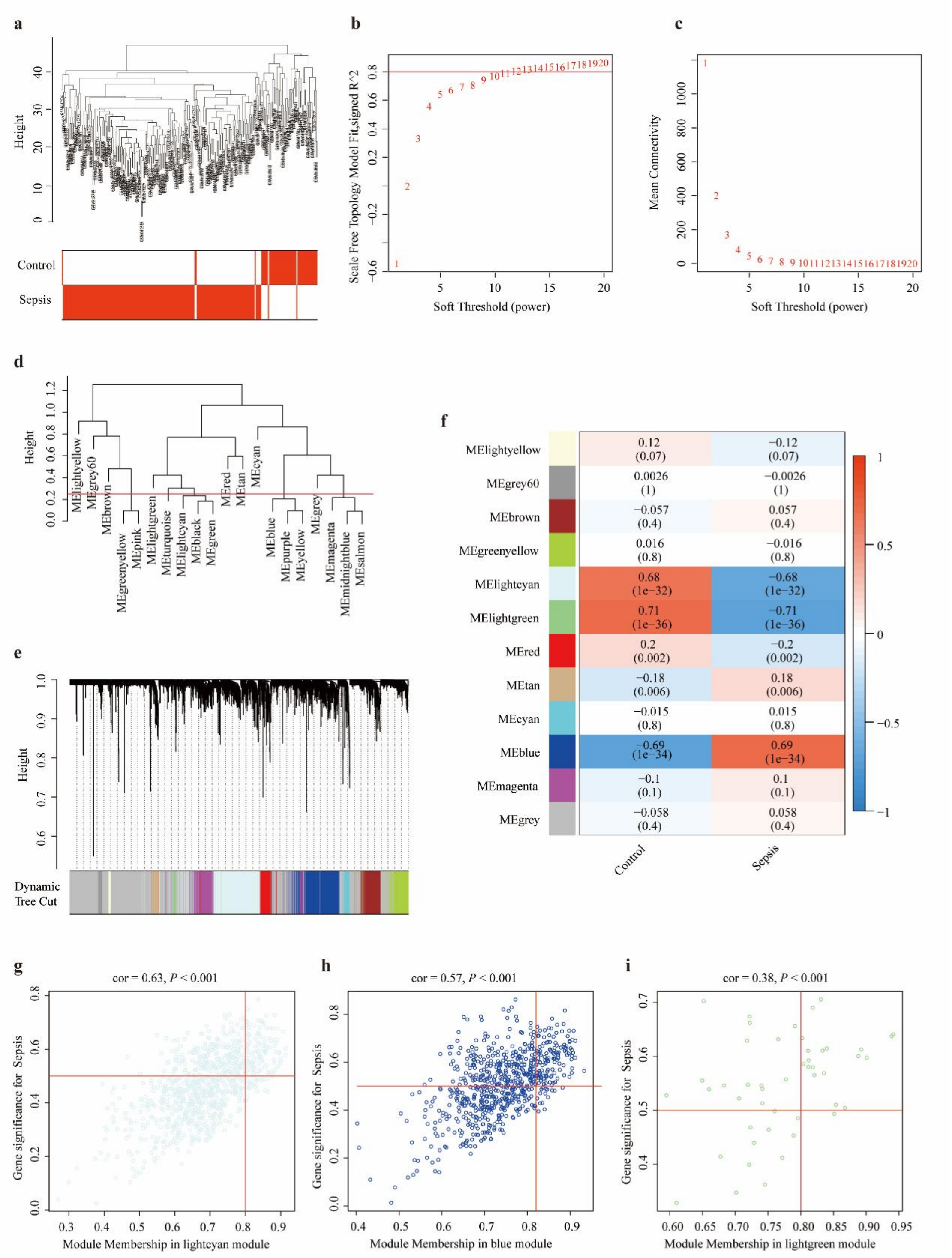

Supplementary Fig. 3. Weighted co-expressionco-expression network related datasets construction in paediatricpediatric sepsis. (a) Clustering dendrogram of 223 samples; (b, c) Analysis of network topology for various soft thresholds (β); (d) Clustering dendrogram of genes; (e) Gene dendrograms obtained by average linkage hierarchical clustering; (f) Module-trait relationships; (g) A scatterplot of GS for recurrence vs. MM in the lightcyan module; (h) A scatterplot of GS for recurrence vs. MM in the blue module; (i) A scatterplot of GS for recurrence vs. MM in the lightgreen module. *GS* gene significance, *MM* module membership.

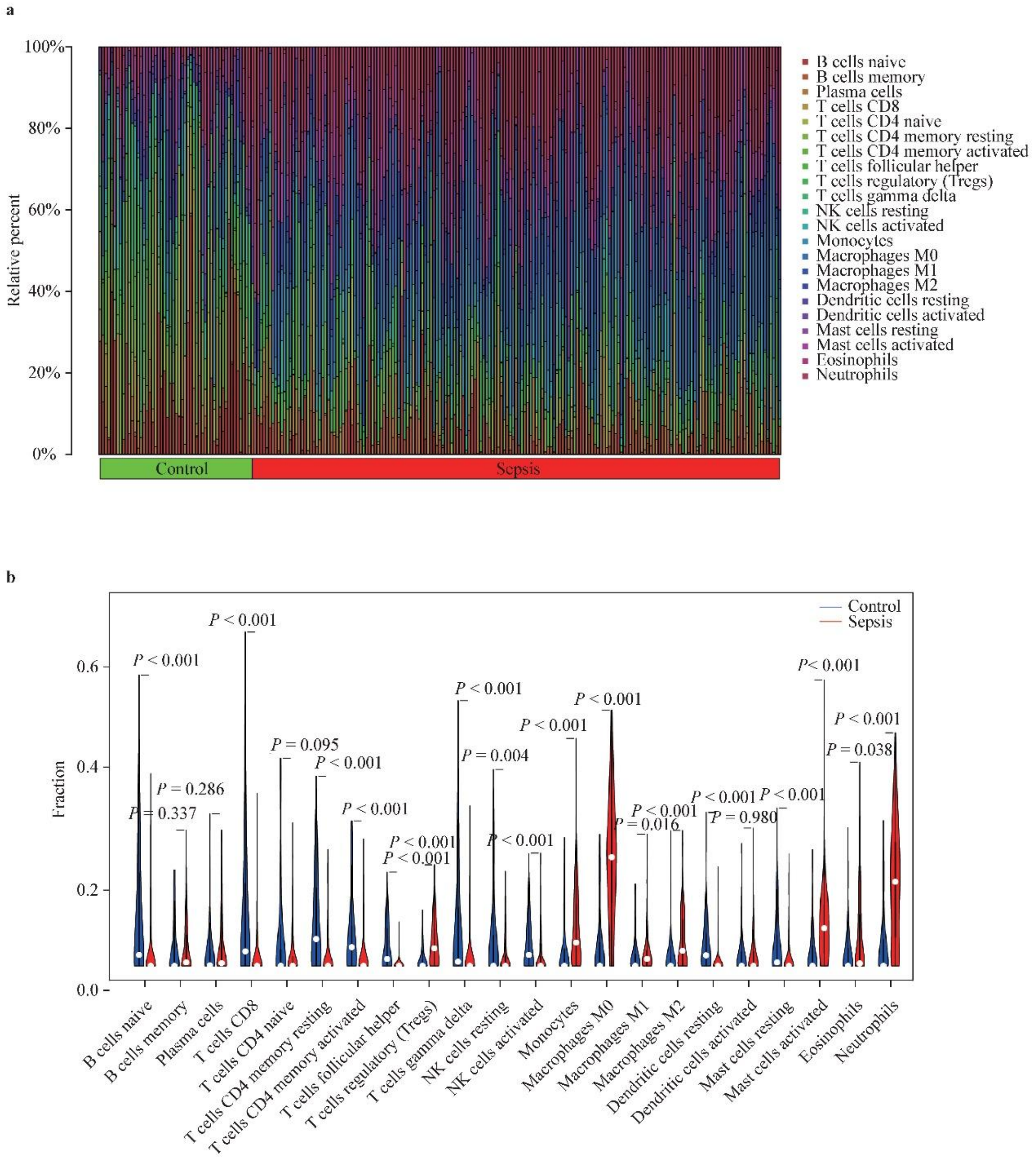

Supplementary Fig. 4. Evaluation and visualization of immune cell infiltration. (a) Boxplot and (b) Violin diagram of the proportion of 22 types of immune cells. *NK* natural killer.

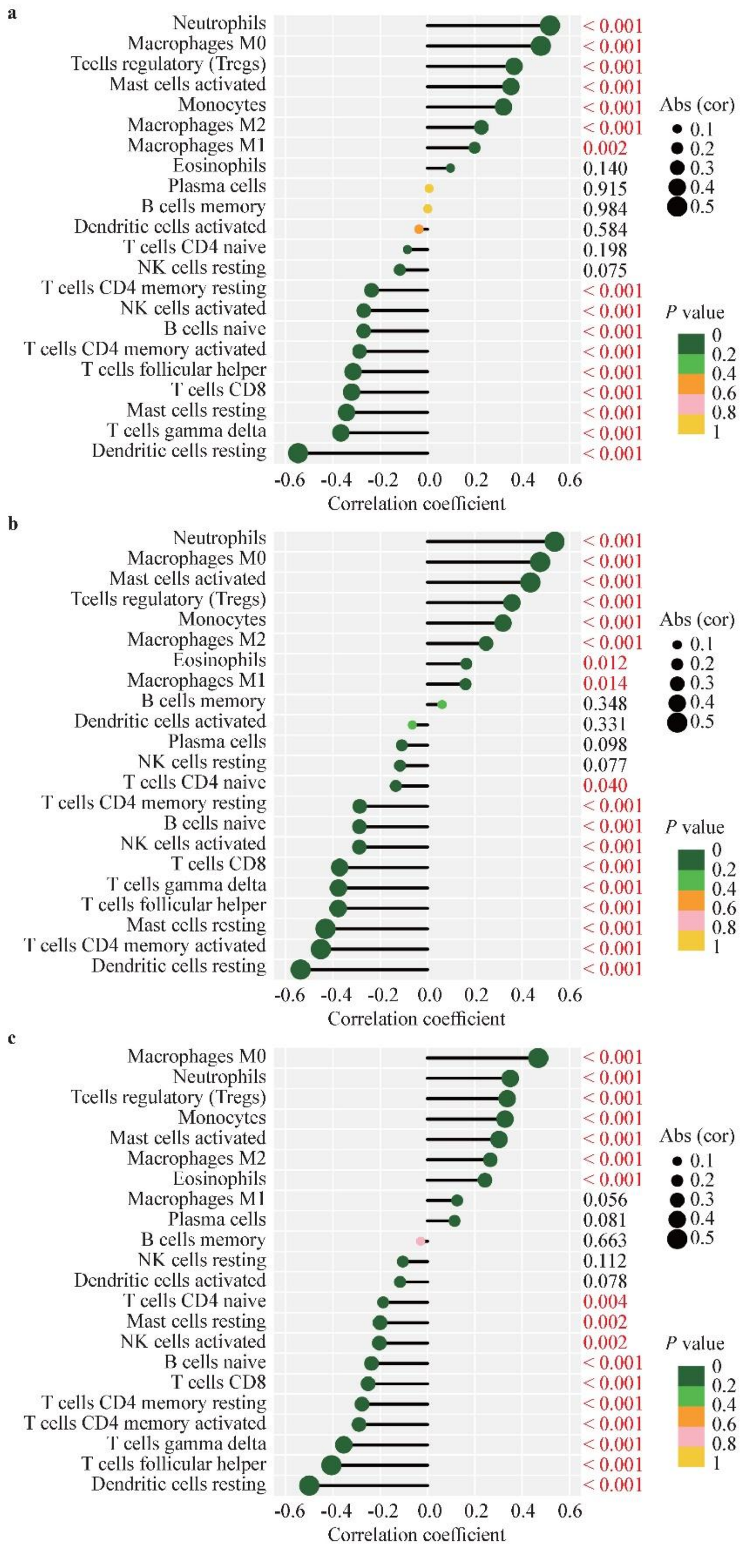

Supplementary Fig. 5. There is a correlation between hub genes and immune cells that have infiltrated the tissue. (a) Correlation between CYSTM1 and infiltrating immune cells; (b) Correlation between MMP8 and infiltrating immune cells; (c) Correlation between CD177 and infiltrating immune cells. *NK* natural killer.
